# Usefulness of 10-2 Matrix Frequency Doubling Technology Perimetry for Detecting Central Visual Field Defects in Preperimetric Glaucoma Patients

**DOI:** 10.1038/s41598-017-15329-1

**Published:** 2017-11-07

**Authors:** Younhea Jung, Hae-Young L. Park, Yi Ryeung Park, Chan Kee Park

**Affiliations:** 1 Department of Ophthalmology and Visual Science, College of Medicine, The Catholic University of Korea, Seoul St. Mary’s Hospital, Seoul, Korea; 2 Seoul St. Mary’s Eye Clinic, Suwon, Korea

## Abstract

It is generally acknowledged that structural loss can precede functional loss in some patients with early glaucoma. However, conventional standard automated perimetry (SAP) has limitations in the detection of functional loss, especially in the macular area. This study explores visual field loss in the macular areas of patients with preperimetric glaucoma exhibiting structural thinning in the area by examining the correlations between the ganglion cell-inner plexiform layer (GCIPL) and the results of matrix frequency-doubling technology (FDT) 10-2 tests. The structure-function relationships between the GCIPL thicknesses and the mean sensitivities (MSs) of the corresponding areas based on conventional SAP 24-2, FDT 10-2, and FDT 24-2 were examined in 62 patients. The highest correlation was found for FDT 10-2 (r = 0.544, *P* < 0.001) followed by FDT 24-2 (r = 0.433, *P* = 0.002) and SAP (r = 0.346, *P* = 0.007). The correlation coefficients between each GCIPL sector and the corresponding central MS according to FDT 24-2 and 10-2 were all statistically significant, and the correlations were significantly stronger for FDT 10-2 than 24-2 in the inferior and inferonasal sectors. In conclusion, preperimetric glaucoma patients with structural loss in the macula as indicated by GCIPL thinning also exhibited functional loss as revealed by FDT 10-2, and the functional loss was less evident with conventional SAP.

## Introduction

Although it is generally considered that glaucomatous field defects start in the periphery and preserve the central field, there are increasing numbers of reports indicating that the macular area is involved even in the early stage of glaucoma^1^. However, standard automated perimetry (SAP), which is conventionally used in clinical practice to determine the functional loss, might not be optimal in the detection of glaucomatous field loss in the macular area because the test points lie 6° apart. Therefore, SAP has only 12 points located in the macular area where over 30% of the retinal ganglion cells (RGCs) are located^1,2^.

We previously reported the ability of short wavelength perimetry (SWAP) 10-2 to detect functional loss in the macular area^3^. However, SWAP 10-2 uses the full threshold strategy, which requires a longer test time and does not provide normative values, and hence does not provide global indices, such as the mean deviation and the pattern standard deviation. These limitations make SWAP 10-2 difficult to apply in general clinical settings.

Frequency-doubling technology (FDT) 10-2 perimetry has an internal normative database and thus provides the mean deviation and pattern standard deviation, which make the results easier to interpret in the clinical setting. FDT 10-2 has several advantages in the detection of functional loss in the macula. First, FDT is based on the frequency-doubling illusion, which occurs when viewing a sinusoidal grating of low spatial frequency undergoing counterphase flickers at a high temporal frequency^4,5^. It has been suggested that FDT represents the function of the magnocellular RGCs with partial input from other RGCs, and several studies have demonstrated that FDT may detect glaucomatous field loss earlier than SAP^6–9^. Second, FDT 10-2 has 32 test points placed 2° apart in the macular area. Therefore, we hypothesized that the structure-function relationship of the macula would be stronger using FDT 10-2.

The purpose of this study was to examine the ability of FDT 10-2 to detect functional loss in the macular area in patients with no evident functional loss as indicated by conventional SAP 24-2 perimetry but with suspected structural loss as indicated by GCIPL thinning.

## Results

A total of 62 patients with preperimetric glaucoma were included in this study. Table 1 presents the baseline characteristics of these patients. The mean age was 71.41 ± 8.95 years, and mean intraocular pressure was 13.64 ± 2.92 mmHg. The mean test durations were 4.56 ± 0.36 min, 5.02 ± 0.26 min, and 4.16 ± 0.14 min for 24-2 SAP, FDT 24-2, and FDT 10-2, respectively.

**Table 1:** Baseline characteristics.

Variable	Mean ± standard deviation
Age (years)	71.41 ± 8.95
Sex (male/female)	26/36
Intraocular pressure (mmHg)	13.64 ± 2.92
Refraction (diopters)	−2.34 ± 2.82
Axial length (mm)	23.92 ± 1.31
Central corneal thickness (μm)	521.54 ± 35.45
Ganglion cell/inner plexiform layer thickness (μm)	
Average	74.24 ± 6.46
Superonasal sector	78.77 ± 7.77
Superior sector	75.59 ± 8.88
Superotemporal sector	76.70 ± 6.43
Inferonasal sector	74.59 ± 9.03
Inferior sector	69.16 ± 10.57
Inferotemporal sector	70.48 ± 10.19
FDT 10-2	
Test duration (min)	4.16 ± 0.14
MD	−3.19 ± 3.28
PSD	3.80 ± 1.95
Centre MS (dB)	27.69 ± 3.61
FDT 24-2	
Test duration (min)	5.02 ± 0.26
MD	−6.43 ± 4.29
PSD	4.12 ± 1.30
Centre MS (dB)	25.61 ± 4.58
SAP 24-2	
Test duration (min)	4.56 ± 0.36
MD	−1.70 ± 1.71
PSD	2.15 ± 1.25
VFI	97.63 ± 2.36
Centre MS (dB)	30.77 ± 2.07

FDT = frequency doubling technology, MD = mean deviation, PSD = pattern standard deviation, MS = mean sensitivity, VFI = visual field index.

Table 2 presents the correlations between the GCIPL thicknesses (GCIPLTs) and the corresponding central mean sensitivities (MSs) in decibels. The correlation coefficients of the average GCIPLTs and the corresponding central MSs (dB) were 0.346 (*P* = 0.007), 0.443 (*P < *0.001), and 0.544 (P < 0.001) for 24-2 SAP, FDT 24-2, and FDT 10-2, respectively. The correlations between each GCIPL sector and the corresponding central MS ranged from 0.212 to 0.602 for FDT 24-2 and from 0.297 to 0.611 for FDT 10-2, and all of these correlations were statistically significant. The correlation coefficients were higher for FDT 10-2 than for FDT 24-2 with marginal significance globally and in the superior and superonasal sectors (*P* = 0.080, 0.071, and 0.070, respectively) and with statistical significance in the inferior and inferonasal sectors (*P* = 0.011 and 0.005, respectively). The correlations between each GCIPL sector and the corresponding central MSs were significant only in the inferotemporal (r = 0.364, *P* = 0.005) and inferior (r = 0.397, *P* = 0.002) sectors for SAP 24-2.

**Table 2:** Pearson correlations between the ganglion cell/inner plexiform layer thicknesses and the corresponding central visual field sensitivities (in decibels).

	SAP 24-2 (dB)		FDT 24-2 (dB)		FDT 10-2 (dB)		P value*
	r	P	r	P	r	P	
GCA average	0.346	0.007	0.443	<0.001	0.544	<0.001	0.080
Superotemporal sector	0.192	0.183	0.212	0.004	0.297	0.021	0.152
Superior sector	0.156	0.246	0.286	0.017	0.431	0.001	0.071
Superonasal sector	0.119	0.365	0.368	0.010	0.455	<0.001	0.070
Inferotemporal sector	0.435	0.001	0.602	<0.001	0.607	<0.001	0.475
Inferior sector	0.392	0.002	0.407	0.004	0.611	<0.001	0.011
Inferonasal sector	0.251	0.053	0.347	0.016	0.567	<0.001	0.005

*P value comparing the difference in the correlation coefficients between FDT 24-2 and FDT 10-2. SAP = standard automated perimetry, FDT = frequency doubling technology, GCA = ganglion cell analysis.

**Figure 1:**
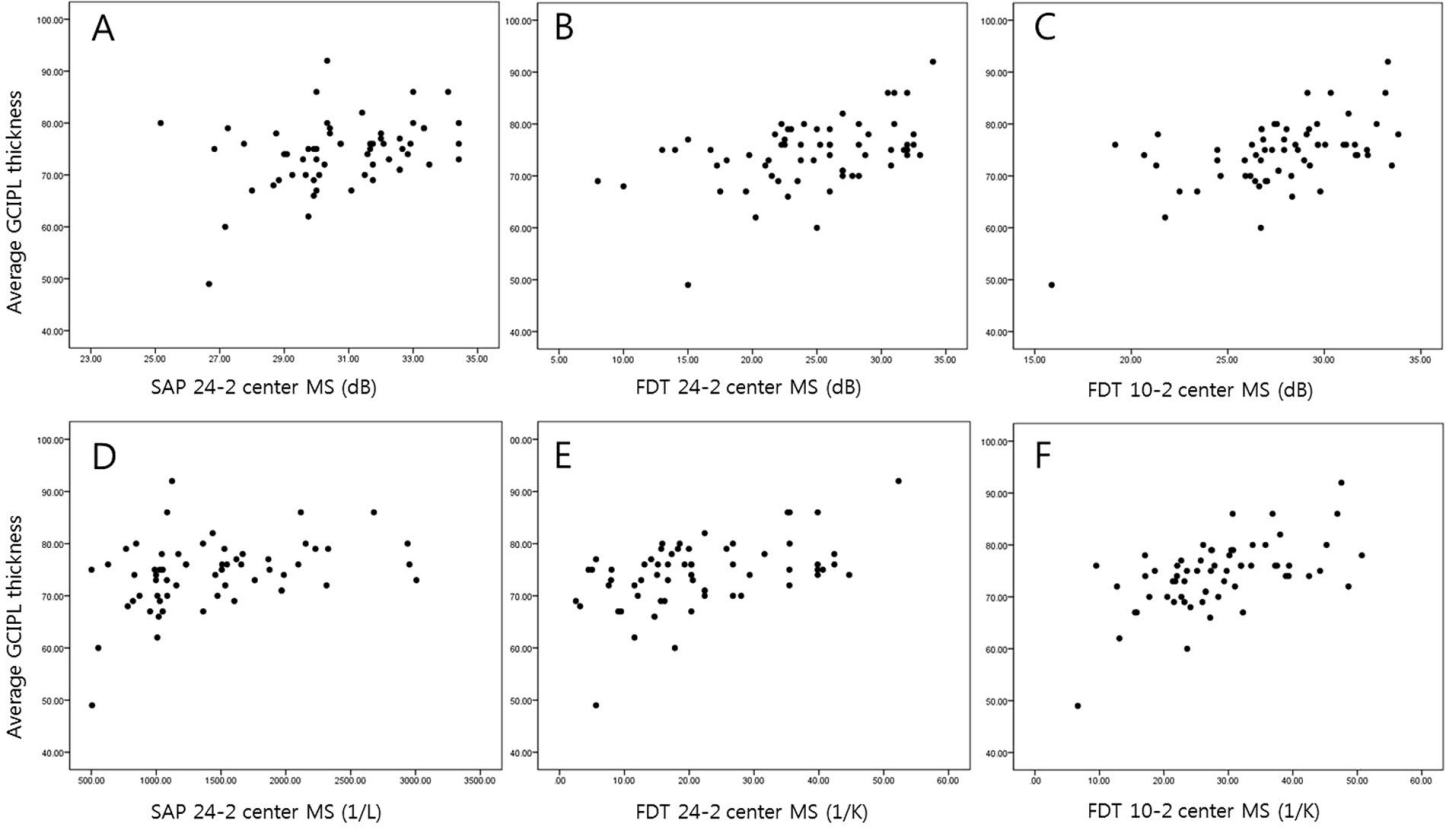
Structure-function relationships of the average ganglion cell/inner plexiform layer thicknesses and standard automated perimetry 24-2, matrix frequency doubling technology 24-2, and matrix frequency doubling technology 10-2 (logged: **A**,**B** and **C**, respectively; unlogged: **D**,**E** and **F**, respectively).

**Figure 2:**
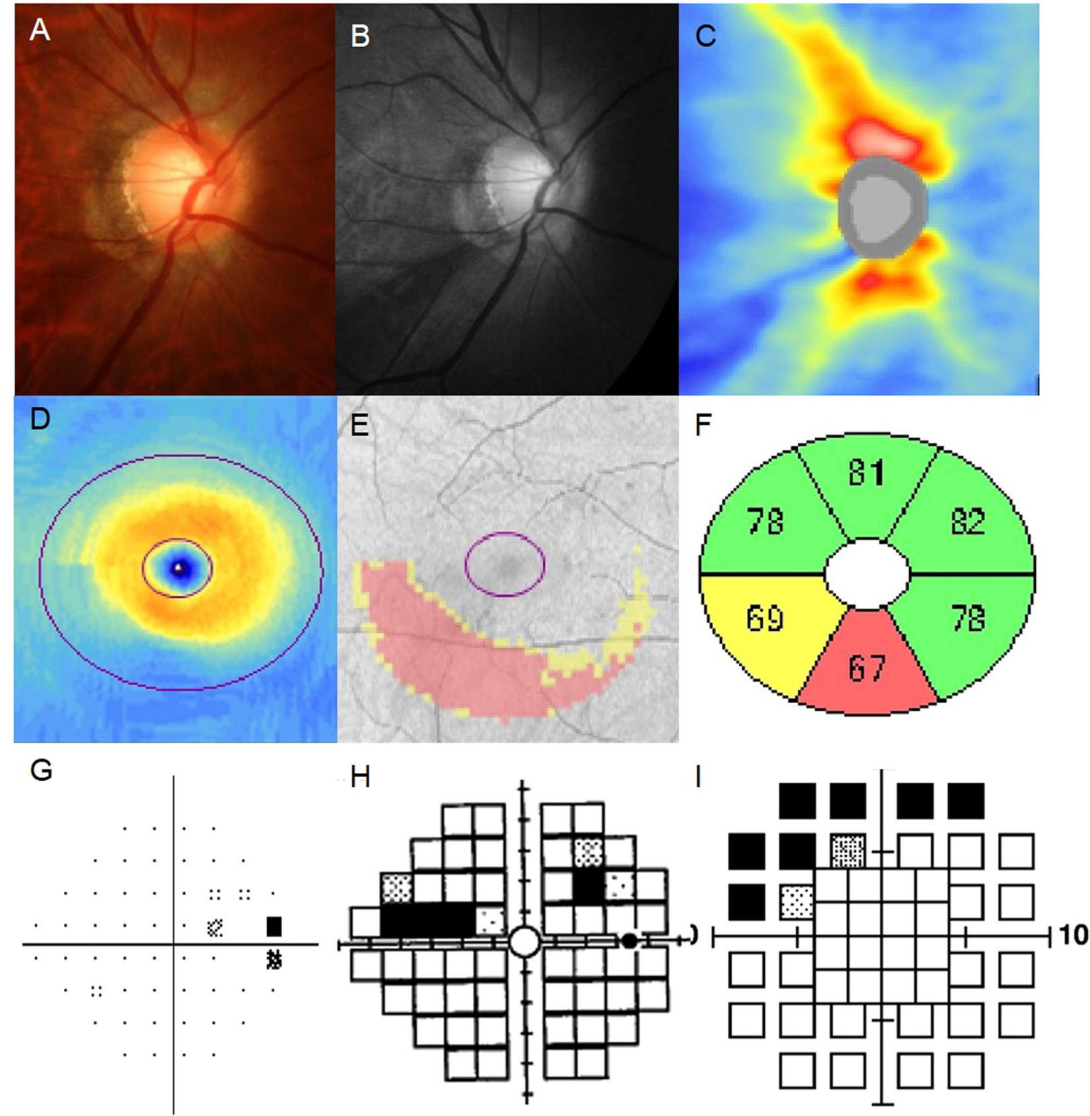
A representative case. A patient with preperimetric glaucoma with thinning of the inferior and inferotemporal sectors in the macular area (**F**) detected by matrix frequency doubling technology 10-2 (**I**) and 24-2 (**H**) but not by standard automated perimetry 24-2 (**G**).

Table 3 presents the correlation between the GCIPLTs and the corresponding central unlogged MSs. Figure 1 provides scatter plots of the GCIPLs and the corresponding central MSs for each perimetry. A representative case of a 55-year-old female with a glaucomatous optic disc with an inferotemporal nerve fibre layer defect is presented in Fig. 2. SAP 24-2 did not reveal central field defects, whereas FDT 24-2 and FDT 10-2 revealed central field defects in the areas in which the GCIPL was thinner.

**Table 3:** Pearson correlations between the ganglion cell/inner plexiform layer thicknesses and the central visual field sensitivities (unlogged).

	SAP 24-2 (1/L)		FDT 24-2 (1/K)		FDT 10-2 (1/K)		P value*
	r	P	r	P	r	P	
GCA average	0.370	0.004	0.492	<0.001	0.563	<0.001	0.190
Superotemporal sector	0.150	0.107	0.215	0.037	0.246	0.045	0.352
Superior sector	0.163	0.226	0.323	0.019	0.435	0.001	0.111
Superonasal sector	0.116	0.377	0.393	0.006	0.415	0.001	0.384
Inferotemporal sector	0.364	0.005	0.525	0.001	0.547	<0.001	0.389
Inferior sector	0.397	0.002	0.484	0.001	0.629	<0.001	0.028
Inferonasal sector	0.386	0.002	0.365	0.011	0.532	<0.001	0.029

*P value comparing the difference in the correlation coefficients between FDT 24-2 and FDT 10-2. SAP = standard automated perimetry, FDT = frequency doubling technology, GCA = ganglion cell analysis.

## Discussion

The purpose of our study was to determine whether reduced macular thickness accompanied visual field loss in preperimetric glaucoma patients. We investigated the relationship between macular thickness as measured by the GCIPL and visual field loss as detected by FDT 10-2 in patients whose visual field loss was not detected by SAP 24-2. To the best of our knowledge, the structure-function relationship of the macular area based on GCIPLT and FDT 10-2 has not been reported in preperimetric glaucoma.

The structure-function relationships between GCIPLTs and mean sensitivities of the macular area as measured by FDT 10-2 were analysed and compared to those of SAP 24-2 and FDT 24-2. Although SAP 24-2, FDT 24-2, and FDT 10-2 all revealed significant correlations with the average GCIPLT, the correlation coefficient was greater for FDT 10-2 than FDT 24-2 with marginal statistical significance. Among the GCIPL sectors, the superior GCIPL sectors did not exhibit significant correlation with the SAP 24-2 results, but significant correlations with the FDT 24-2 and FDT 10-2 results were found. The inferior GCIPL sectors exhibited statistically significant correlations in the inferotemporal and inferior sectors for SAP 24-2 and in all sectors for both FDT 24-2 and FDT 10-2. Furthermore, the correlations were significantly stronger for FDT 10-2 than FDT 24-2 in the inferior and inferonasal sectors.

The better correlations between the FDT 10-2 MSs and the GCIPLTs compared to those for SAP 24-2 can be explained by several factors. First, although there are divergent reports, many studies have reported that FDT perimetry can detect glaucomatous damage earlier than SAP. Racette *et al*.^10^ reported that matrix FDT distinguishes glaucomatous eyes from healthy eyes better than SAP 24-2. Sample *et al*.^11^ also compared the sensitivity of FDT perimetry with that of other perimetries, including SAP, and concluded that FDT is the most sensitive. Liu *et al*.^12^ also reported that matrix FDT perimetry exhibits sensitivity in the detection of glaucoma that is comparable to that of SAP (69% and 68% for matrix FDT and SAP, respectively) and somewhat higher than that of SAP in early glaucoma patients (52% and 46% for matrix FDT and SAP, respectively). In contrast, Burgansky-Eliash *et al*.^13^ and Spray *et al*.^14^ reported comparable performances of FDT 24-2 and SAP 24-2 in the detection of glaucoma. Many have proposed that the superiority of FDT of SAP in the detection of glaucoma is attributable to the reduced redundancy of the M cells that mediate the FDT stimulus, whereas SAP is not specific to a particular RGC type^15^. Hence, the relatively small proportion and reduced overlap of the receptive fields of M cells in the macular area may account for the difference between FDT and SAP. However, Swanson *et al*.^16^ reported that the M cell pathway is more sensitive to SAP than FDT and suggested that the difference between FDT and SAP may be due to the larger stimulus size and smaller contrast range of the FDT stimuli.

Second, the test points of FDT 10-2 are denser in the macular area than those of SAP 24-2. FDT 10-2 has 32 test points in the macular region, whereas the SAP 24-2 has only 12 points. Hangai *et al*.^17^ reported several cases in paracentral visual field defects were detected only with SAP 10-2 and not with SAP 24-2.

Additionally, different threshold strategy algorithms (SITA vs. ZEST) could also account for this difference. In this study, the SITA and ZEST algorithms, which are both widely used in clinical practice, were used for SAP and matrix FDT, respectively. SITA and ZEST are both Bayesian perimetric strategies based on maximum-likelihood principles, but SITA is a mixture of both maximum-likelihood threshold and staircase procedures, whereas ZEST is based on the maximum-likelihood test procedure^18^. Turpin *et al*.^18^ compared the performances of a SITA-like strategy, the staircase-QUEST, and ZEST using a computer simulation model and reported that ZEST outperformed the staircase-QUEST in terms of producing lower mean errors.

Only subjects with preperimetric glaucoma were included in the present study; therefore, one could argue that a few of these patients could have exhibit defects with FDT but not SAP, which could have resulted in selection bias. However, in our study, we examined the correlation between the GCIPL and each perimetry rather than the diagnostic capabilities.

We previously reported that SWAP 10-2 can detect functional damage in the macular area better than SAP 24-2^3^. However, the longer test duration and the difficulty of the interpretation of the data limits the usefulness of SWAP 10-2 in clinical settings. Additionally, van der Schoot *et al*.^19^ reported that SWAP does not detect conversion to glaucoma earlier than SAP. Further research comparing various perimetries using 10-2 strategies may provide more information about the visual function of the macular area. Additionally, the current study is a retrospective study that evaluated the usefulness of FDT 10-2 in the detection of functional loss in the macular area, and further longitudinal studies are warranted to compare the usefulness of various visual field strategies in the detection of the progression of the disease in this area.

In conclusion, this study found significant correlations between reduced GCIPLTs and the mean sensitivities of the corresponding areas as obtained by FDT 10-2, FDT 24-2, and SAP 24-2 in preperimetric glaucoma patients. However, the FDT 10-2 results produced the best correlations with the GCIPLTs. These findings suggest the usefulness of FDT 10-2 in patients with reduced GCIPLTs even if their visual fields seem normal on SAP 24-2.

## Methods

The present study included 62 preperimetric glaucoma patients who visited the Seoul St. Mary’s Hospital College of Medicine of the Catholic University of Korea between December 2015 and February 2016. The study was conducted in accordance with the ethical standards of the Declaration of Helsinki and was approved by the Institutional Review Board of Seoul St. Mary’s Hospital College of Medicine, which waived the written informed consent because of the study’s retrospective design.

For each patient, the following examinations were conducted, and the results were analysed: best-corrected visual acuity, gonioscopy, dilated fundoscopic examination, fundus photography, red-free fundus photography, Goldmann applanation tonometry, achromatic SAP 24-2 using the SITA standard program (Humphrey Visual Field Analyzer, Carl Zeiss Meditec, Inc., Dublin, CA), FDT 24-2 perimetry and FDT 10-2 perimetry using the FDT Humphrey Matrix (Carl Zeiss Meditec, Inc.), and spectral domain (SD) optical coherence tomography (OCT) scanning (Cirrus HD-OCT 4000, software version 6.5.0.772; Carl Zeiss Meditec, Inc.) including ganglion cell analysis (GCA). All patients underwent SAP testing at least twice prior to enrolment.

The following were required for inclusion in the study: a best-corrected visual acuity of at least 20/40, a spherical error between +4.0 and −6.0 diopters, a cylinder error within ±2 diopters, an open angle on gonioscopy, an intraocular pressure (IOP) ≤21 mmHg with or without glaucoma medication, and a reliable GCA with at least one sector coded as yellow (outside 95% of the built-in normative database) or red (outside 99% of the built-in normative database), reliable visual field (VF) test results with false-positive and -negative error rates <15% and a fixation loss <15% for both SAP and FDT. A normal SAP 24-2 required the pattern standard deviation of a *P* value >5%, and the glaucoma hemifield test results to be within normal limits.

The exclusion criteria included the following ocular or systemic conditions that might affect the VF results: any history of retinal disease; neurological disease; cerebrovascular disease; medications that might affect the VF, such as plaquenil; ocular trauma; and ocular surgery other than uncomplicated cataract surgery. When both eyes met the inclusion criteria, one eye of each subject was chosen randomly.

Preperimetric glaucoma was defined by a normal SAP 24-2 in addition to glaucomatous optic neuropathy (e.g., neuroretinal rim thinning, notching, and/or an RNFL defect) based on the colour and red-free fundus photography as detected independently by two glaucoma specialists who were blinded to the patient data (HP and CP). Disagreements between the two specialists were resolved by consensus.

### Cirrus HD-OCT Imaging

Only Cirrus HD-OCT images with signal strengths ≥6 were used. Scans exhibiting algorithm segmentation failure, any blinking artefacts, involuntary ocular movement, or any misalignment were excluded. For GCA, the 512 × 128 cube scan mode was used to obtain the three-dimensional macular cube OCT scan. The GCA algorithm automatically measured the macular GCIPLT, which included the ganglion cell layer and the inner plexiform layer (IPL). The average GCIPLT and six sectoral (i.e., superonasal, superior, superotemporal, inferotemporal, inferior, and inferonasal) GCIPLTs were measured within a macular elliptical annulus with vertical inner and outer diameters extending from 1.0 mm to 4.0 mm respectively, and horizontal inner and outer diameters extending from 1.2 mm to 4.8 mm, respectively^20^.

### Visual Field Examination

All patients underwent SAP 24-2 using the Humphrey Field Analyzer and FDT 24-2 and FDT 10-2 using the FDT Humphrey Matrix. All VF tests were performed within 3 months, and the order of the VF tests was randomized using a random table. SAP 24-2 was performed using the Swedish interactive threshold algorithm (SITA) with a stimulus size of 0.43°(Goldmann size III) placed 6° apart. Both FDT 24-2 and FDT 10-2 used the Matrix threshold test algorithm known as the Zippy Estimation of Sequential Thresholds (ZEST), which is comparable to SITA^21^. FDT 24-2 was performed with a stimulus of 5°, a spatial frequency of 0.5 cycles/degree, and a temporal frequency of 18 Hz. FDT 10-2 was performed using a stimulus of 2°, a spatial frequency of 0.5 cycles/degree, and a temporal frequency of 12 Hz.

The structure-function relationships were compared between the GCIPLTs and MSs of the topographically corresponding areas as measured with 24-2 SAP, FDT 24-2, and FDT 10-2. VF sensitivity was analysed using both the logarithmic decibel scale and the nonlogarithmic (1/Lambert and 1/Michelson contrast in SITA and FDT, respectively) scale. The nonlogarithmic value was calculated as decibels = 10 × log(1/Lambert) for SAP and as decibels = 20 × log(1/Michelson contrast) for FDT.

**Figure 3:**
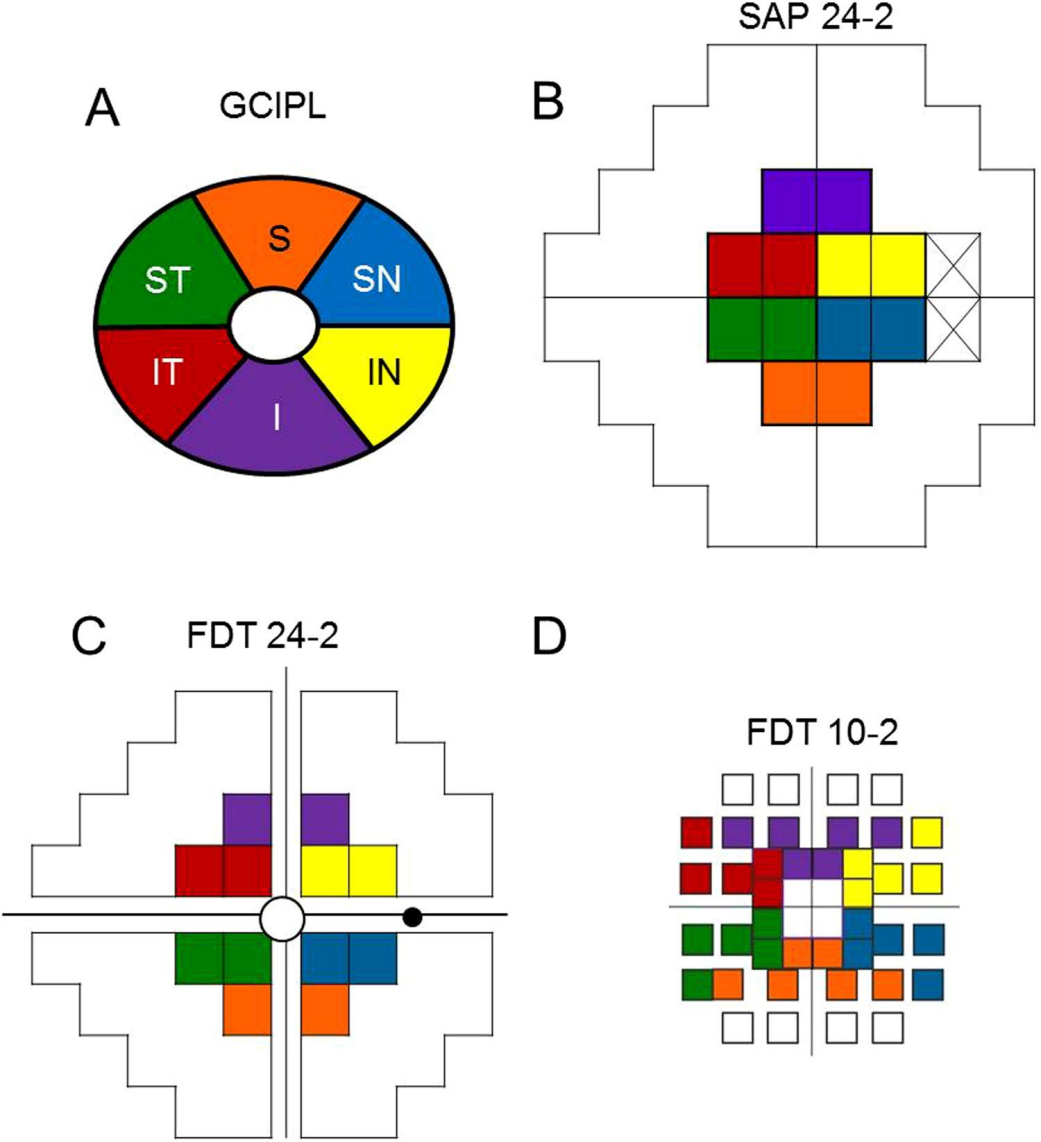
Ganglion cell/inner plexiform layer sectors (**A**) and the corresponding visual field areas in standard automated perimetry 24-2 (**B**), matrix frequency doubling technology 24-2 (**C**), and matrix frequency doubling technology 10-2 (**D**).

The SAP 24-2 central MS was defined as the average of the data from the 12 central points, which were assumed to topographically correspond to the macular area scanned in the GCA^20^. Similarly, the FDT 24-2 central MS and FDT 10-2 central MS were defined as the average of the data from the 12 central points and 32 central points, respectively, for each perimetry (Fig. 3)^3,22^. After taking RGC displacement in the foveal area into account, the foveal sensitivity values at the centres of the FDT 24-2 and FDT 10-2 were excluded from the analyses. VF sensitivity and GCIPL data from the sectors from the left eyes were converted into the right-eye format.

### Statistical Analyses

To assess structure-function relationships, regression analyses were performed while treating the average/sectoral GCIPLTs as the independent variables and the topographically corresponding sensitivities of each perimetry as the dependent variables. Pearson’s correlation coefficients were analysed to examine the associations between the average/sectoral GCIPLTs and the sensitivities of the corresponding areas of each perimetry.

The relationships between the average GCIPLTs and the central MSs were analysed for each perimetric modality and strategy. Additionally, the relationship between each GCIPL sector and central MS of each topographically corresponding sector were also analysed. Differences in the correlation coefficients were compared using the method described by Steiger^23^. A *P* value of less than 0.05 was considered statistically significant. The Statistical Package for the Social Sciences (SPSS, Inc., Chicago, IL, software version 18.0) was used for the statistical analyses.
